# Regulation of Bile Acid and Cholesterol Metabolism by PPARs

**DOI:** 10.1155/2009/501739

**Published:** 2009-07-14

**Authors:** Tiangang Li, John Y. L. Chiang

**Affiliations:** Department of Integrative Medical Sciences, Northeastern Ohio Universities College of Medicine, Rootstown, OH 44272, USA

## Abstract

Bile acids are amphipathic molecules synthesized from cholesterol in the liver. Bile acid synthesis is a major pathway for hepatic cholesterol catabolism. Bile acid synthesis generates bile flow which is important for biliary secretion of free cholesterol, endogenous metabolites, and xenobiotics. Bile acids are biological detergents that facilitate intestinal absorption of lipids and fat-soluble vitamins. Recent studies suggest that bile acids are important metabolic regulators of lipid, glucose, and energy homeostasis. Agonists of peroxisome proliferator-activated receptors (PPAR*α*, PPAR*γ*, PPAR*δ*) regulate lipoprotein metabolism, fatty acid oxidation, glucose homeostasis and inflammation, and therefore are used as anti-diabetic drugs for treatment of dyslipidemia and insulin insistence. Recent studies have shown that activation of PPAR*α* alters bile acid synthesis, conjugation, and transport, and also cholesterol synthesis, absorption and reverse cholesterol transport. This review will focus on the roles of PPARs in the regulation of pathways in bile acid and cholesterol homeostasis, and the therapeutic implications of using PPAR agonists for the treatment of metabolic syndrome.

## 1. Introduction

Fibrates have been used for decades to treat hypertriglyceridemia or mixed hyperlipidemia for their ability to significantly reduce plasma triglyceride levels [1]. Fibrate treatments also modestly elevate plasma HDL-C and slightly decrease plasma LDL-C. Studies in the past have revealed that the hypolipidemic effects of fibrates are mainly a result of activation of the nuclear receptor peroxisome proliferator-activated receptor *α* (PPAR*α*), which belongs to the nuclear hormone receptor superfamily [2]. PPAR*α* can be activated by natural fatty acids and fibrates. PPAR*α* forms a heterodimer with nuclear receptor RXR and recognizes a consensus PPAR responsive element (PPRE) on its target gene promoters. PPAR*α* regulates a network of genes that promote lipolysis and fatty acid *β*-oxidation, the major mechanisms mediating the lipid lowering effects of fibrates. Based on the sequence homology, two additional PPAR isoforms were identified and named PPAR*γ* and PPAR*β*/*δ* [3, 4]. PPAR*γ* plays critical roles in adipocyte differentiation, lipid storage, inflammation, and energy metabolism. PPAR*γ* is activated by the thiazolidinediones (TZDs) drugs, which improve insulin sensitivity and lower plasma glucose levels in diabetes [1]. PPAR*δ* plays a role in fatty acid and energy metabolism in the muscle. Activation of PPAR*δ* has been shown to prevent dyslipidemia and obesity in animal models of metabolic syndromes [5, 6]. PPAR agonists have been extensively investigated for their therapeutic benefits in improving diabetes, dyslipidemia, and features of metabolic syndromes. 

Bile acids are amphipathic molecules derived from cholesterol in the liver [7, 8]. Bile acid synthesis generates bile flow from the liver to the intestine. The transport of bile acids between liver and intestine is referred to as the enterohepatic circulation of bile, which plays important roles in liver function, liver physiology, and metabolic regulation. Bile acids are detergent molecules that facilitate biliary excretion of cholesterol, endogenous metabolites and xenobiotics, and intestinal absorption of lipids and nutrients. In cholestatic liver diseases, bile acids accumulate at high concentrations in the liver, resulting in hepatocyte injury, impaired liver function, fibrosis and cirrhosis. The liver plays a central role in maintaining cholesterol homeostasis by balancing multiple pathways including de novo cholesterol and bile acid synthesis, dietary cholesterol uptake, biliary cholesterol excretion, lipoprotein synthesis, and reverse cholesterol transport. Defects in bile acid synthesis due to mutations in bile acid biosynthetic genes caused both abnormal cholesterol metabolism and bile acid metabolism, which led to cholesterol gallstone disease, dyslipidemia, and cardiovascular diseases in humans [9]. This review will summarize the recent development in understanding the role of PPARs in regulation of bile acid and cholesterol homeostasis, and the therapeutic implications in using PPAR agonists for treating metabolic dyslipidemia and reducing the risk of cardiovascular and heart diseases.

## 2. Bile Acid Synthesis and Transport

### 2.1. Bile Acid Synthesis

**Figure 1:**
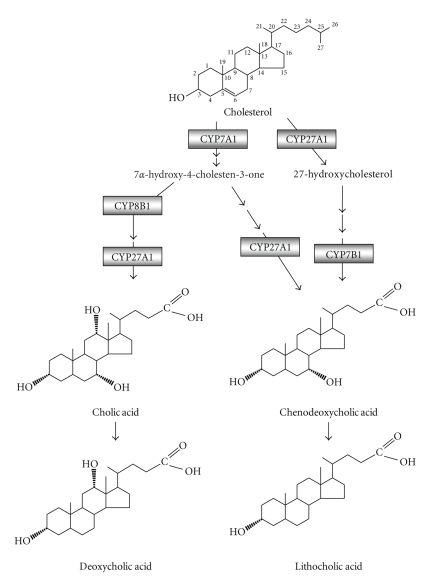
*Bile acid synthesis. * Bile acids are synthesized from cholesterol in the liver through two pathways: the classic pathway and the alternative pathway. In human liver, bile acid synthesis mainly produces two primary bile acids, cholic acid (CA), and chenodeoxycholic acid (CDCA). Key regulatory enzymes in both pathways are indicated. CYP7A1 catalyzes the first the rate-limiting step in the classic pathway to convert cholesterol into 7*α*-hydroxycholesterol, while CYP27A1 initiates the alternative pathway by converting cholesterol into 27-hydroxycholesterol, which is then 7*α*-hydroxylated by oxysterol 7*α*-hydroxylase (CYP7B1). CYP8B1 regulates the cholic acid synthesis in the classic pathway. In the intestine, primary bile acid CA and CDCA are dehydroxylated at the 7*α*-position by the bacterial enzymes to produce the secondary bile acids, deoxycholic acid (DCA), and lithocholic acid (LCA), respectively.

In humans, bile acid pool consists of primary bile acids (cholic acid, CA, and chenodeoxycholic acid, CDCA) and secondary bile acids (deoxycholic acid, DCA, and lithocholic acid, LCA) [7]. Primary bile acids are synthesized from cholesterol exclusively in the liver through two general pathways, the classic pathway and the alternative pathway (Figure 1) [9]. Secondary bile acids are derived from primary bile acids in the intestine by bacterial enzymes. Enzymes that catalyze these multistep reactions are located in the endoplasmic reticulum, mitochondria, cytosol, and peroxisomes. The classic pathway is also known as the neutral pathway for most of its intermediates are neutral sterols. In human, this pathway produces CA and CDCA in approximately equal amounts. Cholesterol 7*α*-hydroxylase (CYP7A1), a microsomal cytochrome p450 enzyme, catalyzes the first and rate-limiting step in the classic pathway to convert cholesterol into 7*α* hydroxycholesterol [10]. Microsomal 3*β*-hydroxy-27-steroid dehydrogenase/isomerase (3*β*-HSD) then converts 7*α*-hydroxycholesterol into 7*α*-hydroxy-4-cholestene-3-one, which is the common precursor for both CA and CDCA. 7*α*-hydroxy-4-cholestene-3-one can be hydroxylated at C-12 position by microsomal sterol 12*α*-hydroxylase (CYP8B1) and modified by other enzymes and eventually converted to CA. Alternatively, without 12*α*-hydroxylation, 7*α*-hydroxy-4-cholestene-3-one is converted to CDCA. Mitochondrial sterol 27-hydroxylase (CYP27A1) mediates the steroid side chain oxidation and cleavage to give carboxyl groups in both CA and CDCA synthesis [11]. The alternative pathway, also called the acidic pathway, was originally revealed by the identification of several acidic intermediates which are not present in the classic pathway [12, 13]. The alternative pathway mainly produces CDCA. CYP27A1 catalyzes the first two steps and converts cholesterol into 27-hydroxycholesterol and then 3*β*,7*α*-dihydroxy-5-cholestenoic acid [14]. Oxysterol 7*α*-hydroxylase (CYP7B1) then catalyzes the hydroxylation reaction at C-7 position of these two intermediates, which are subsequently converted to CDCA by the same enzymes in the classic pathway. In humans, the classic pathway is thought to be the major bile acid biosynthesis pathway in normal physiology in humans. 

### 2.2. Bile Acid Transport

#### 2.2.1. Enterohepatic Circulation

**Figure 2:**
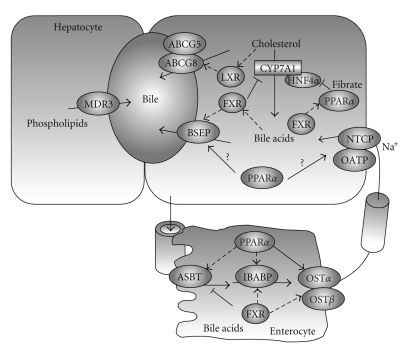
*Enterohepatic circulation of bile. * Bile acids are transported across the canalicular membrane of hepatocytes by BSEP. Cholesterol is either converted into bile acids for biliary secretion or transported by ABCG5/G8 into the bile. MDR3 mediates biliary phospolipids secretion. Cholesterol, bile acids and phospholipids form mixed micelles to solublize cholesterol and reduce bile acid cytotoxicity. After food intake, gallbladder contracts and releases bile acids into intestine. Approximately 95% of bile acids are reabsorbed into the enterocytes. OST*α*/OST*β* heterodimeric transporter mediates basolateral bile acid efflux into the portal circulation. NTCP and OATPs mediate hepatic basolateral uptake of bile acids, which are then resecreted into the bile. In the hepatocytes, bile acid-activated FXR feedback inhibits CYP7A1 and NTCP, and thus bile acid synthesis and uptake. Bile acid-activated feed-forward stimulates BSEP and biliary bile acid secretion. Cholesterol derivatives oxysterols activate LXR, which induces ABCG5/G8 and biliary cholesterol secretion. In the intestine, FXR inhibits ASBT and stimulates IBABP and OST*α* and OST*β*.

Bile acids, once produced in the liver, are transported across the canalicular membrane of the hepatocytes into the bile and stored in the gallbladder. After each meal, bile acids are released into the intestinal tract, efficiently reabsorbed in the ileum, and transported back to the liver via portal blood for reexcretion into the bile. This process is referred to as enterohepatic circulation of the bile (Figure 2) [8]. Bile acid transporters play important roles in this transport process [15]. The biliary excretion of bile acids is the major driving force of bile flow. The bile acid pool size is defined as the total amount of bile acids circulating in the enterohepatic circulation. In humans, bile acid pool consists of CA, CDCA, and DCA in an approximate 40 : 40 : 20 ratio, with a mass of around 2.5–3 gm. After reaching the small intestine, approximately 95% of the bile acids are reabsorbed and only 5% is lost into the feces. The daily loss of bile acids is compensated by de novo synthesis in the liver and thus, a constant bile acid pool is maintained.

#### 2.2.2. Hepatic Bile Acid Transport

Hepatocytes are polarized epithelial cells with basolateral (sinusoidal) and apical (canalicular) membrane domains. Hepatocytes take up bile acids through the basolateral membrane, which is in direct contact with the portal blood plasma, and excrete bile acid at the canalicular membrane into the bile [16]. Bile acids are conjugated with taurine or glycine in the peroxisomes and present as bile salts. They cannot cross the cell membrane and need active transport mechanisms for cellular uptake [17]. Two bile acid transporters, Na^+^-dependent taurocholate transporter (NTCP) and organic anion transporters (OATPs) are responsible for basolateral bile acid transport into the hepatocytes (Figure 2). The NTCP cotransports two Na^+^ down its gradient into the hepatocytes along with one molecule of conjugated bile acid [18]. Na^+^-dependent bile salt uptake pathway accounts for 80% of the total taurocholate uptake and is considered as the major bile acid transport system located at the basolateral membrane [19]. The Na^+^-independent bile salt uptake is mediated by several members of OATP family. These transporters have wide substrate preference. Besides conjugated and unconjugated bile acids, many amphipathic organic compounds such as bilirubin, selected organic cations and numerous drugs are also taken up by these transporters [20]. In rat liver, Oatp-1, -2, and -4 account for the bulk Na^+^-independent bile acid uptake while OATP-C is the most relevant isoform in humans [21–24].

Since the biliary bile acids concentration is about 100 to 1000 fold higher in the bile than in the hepatocytes, canalicular bile acid transport represent the rate-limiting step in bile formation. Several members of the ATP-binding cassette (ABC) transporter family are responsible for transporting bile acids and other organic compounds across the canalicular membrane against their concentration gradients. The bile salt export pump (BSEP, ABCB11), originally identified as the sister of P-glycoprotein (SPGP), is mainly responsible for bile acid transport at the canalicular membrane (Figure 2) [25]. Mutations in *BSEP * were first identified in patients with progressive familial intrahepatic cholestasis subtype 2 (PFIC-2). The absence of functional BSEP in the hepatic canalicular membrane and less than 1% of normal biliary bile acid concentration found in these patients suggested that BSEP is the major canalicular bile acid transport system [26]. 

After bile acids are pumped into the bile, they stimulate phospholipids and cholesterol secretions into the bile, followed by passive inflow of water [27]. Phospholipids are excreted via the phospholipid flippase MDR3 (ABCB4), and the major phospholipid in the bile is phosphatidylcholine [28, 29]. Biliary free cholesterol secretion mediated by ABCG5/G8 transporters is an important route for hepatic cholesterol elimination. Mice lacking ABCG5 and ABCG8 showed decreased biliary cholesterol concentration, while transgenic expression of ABCG5 and ABCG8 in mice resulted in increased biliary cholesterol secretion [30]. Bile acids, phospholipids, and cholesterol are three major organic solutes of the bile and once secreted, they form mixed micelles to increase cholesterol solubility and reduce their toxicity to the bile duct. Normal bile formation depends largely on balanced secretion of these constituents. Impaired secretions will disrupt the bile flow and result in cholestasis or cholesterol gallstone disease.

#### 2.2.3. Intestine Bile Acid Transport

In the intestine lumen, bile acids are reabsorbed mostly at the terminal ileum. Like the hepatic basolateral uptake system, intestinal bile acid uptake is also mainly mediated by the apical sodium-dependent bile salt transporter (ASBT) [31]. This transporter has substrate specificity for both primary and secondary conjugated and unconjugated bile acids. Unlike some hepatic bile acid transporters that also mediate the secretion of other organic compounds, the substrates for ASBT is strictly limited to bile acids [32]. 

Once absorbed into the enterocytes, bile acids bind the intestinal bile acid binding protein (I-BABP) and are transported to the basolateral membrane for secretion (Figure 2) [33]. Recently identified heterodimeric organic solute transporters OST*α*/OST*β* appeared to be the major basolateral bile acid transport system in the intestine and many other epithelial cells [34]. This is supported by studies showing in mouse that overexpression of OST*α* and OST*β* enhanced basolateral efflux of taurocholate, while mice lacking *Ostα* showed marked decreases in intestinal bile acid absorption, serum bile acid concentration, and bile acid pool size [35].

## 3. PPAR Regulation of Bile Acid Synthesis and Transport

### 3.1. PPAR Regulation of Bile Acid Synthesis

Early clinical studies have found consistent increases in biliary cholesterol saturation and the risk of cholesterol gallstone formation in hyperlipidemic human patients following long-term fibrate therapies [36–39]. Despite the observed decrease in plasma LDL-C and increase in plasma HDL-C by fibrate treatments, biliary cholesterol secretion was found to be increased in both normal and hyperlipidemic individuals after fibrate treatments. Biliary bile acid secretion was also reported to be decreased by fibrates [36, 38]. In contrast, biliary phospholipid secretion, which may also affect normal bile flow and cholesterol gallstone formation, seemed to be unaffected [38, 39]. Fibrate treatment has been found to associate with decreased CYP7A1 mRNA expression and enzyme activity. In one study, bezafibrate reduced hepatic CYP7A1 enzyme activity by 60% in normolipidemic gallstone patients [40]. In another study, both gemfibrozil and bezafibrate reduced the rate of cholesterol 7*α*-hydroxylation by 55% in patients with hypercholesterolemia [41]. Human with genetic defects in *CYP7A1 * developed premature hypercholesterolemia and gallstone disease [42]. It is likely that inhibition of hepatic CYP7A1 activity following long-term fibrate treatments may account for the reduced cholesterol catabolism and bile acid output, leading to imbalanced bile acid and cholesterol secretion, increased biliary cholesterol saturation, and the incidence of cholesterol gallstone formation.

**Figure 3:**
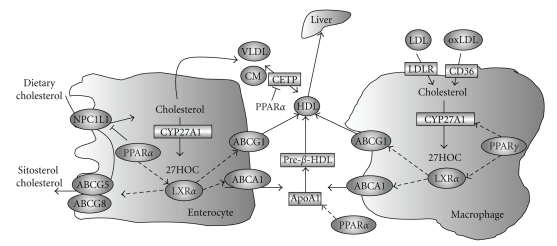
*Reverse cholesterol transport. * In the intestine, dietary uptake of cholesterol is mediated by NPC1L1. ABCG5/G8 effluxes sitosterols and cholesterol back to the intestine lumen and limits intestinal sterol absorption. Oxysterols activate LXR, which induces ABCA1 and ABCG1 to transport cholesterol to ApoA1 and HDL, respectively. PPAR*α* activation reduces NPC1L1 and fractional cholesterol absorption, and may promote cholesterol secretion by stimulating CYP27A1 and LXR activation of ABCA1 and ABCG1. In macrophages, LDLR and CD36 mediate LDL and oxidized-LDL uptake, respectively. CYP27A1 converts cholesterol into 27-hydroxycholesterol, which may activate LXR and cholesterol efflux via ABCA1 and ABCG1. Cholesterol can also be secreted in the form of 27-hydroxycholesterol. PPAR*γ* induces CYP27A1 and LXR, and positively regulates the cholesterol efflux from macrophages. PPAR*α* induces ApoA1 and inhibits CETP, and thus increases circulating HDL-C levels.

Consistent with these observations, studies performed in cell-based models or animal models revealed that fibrate inhibition of hepatic CYP7A1 activity might be a result of PPAR-dependent repression of *Cyp7a1 * transcription. Using cell-based gene reporter assays, two groups showed that PPAR*α*/RXR and Wy14643 repressed both human and rat *CYP7A1 * promoter reporter activities [43, 44]. Although a putative PPRE was mapped in the human *CYP7A1 * promoter, this site did not bind PPAR*α*/RXR, but was a previously identified binding site for nuclear receptor HNF4*α*, a positive regulator of *Cyp7a1 * transcription. The mechanistic studies further revealed that PPAR*α* inhibited *Cyp7a1 * by decreasing the cellular levels of HNF4*α*. Consistent with these in vitro studies, ciprofibrate treatment was shown to inhibit CYP7A1 mRNA expression and enzyme activity in both rat and mouse livers in vivo [45]. Although various fatty acids may act as the endogenous PPAR*α* agonists, it seems that PPAR*α* may not play a critical role in controlling CYP7A1 gene expression under normal physiology, as genetic knockout of *Pparα* in mice did not affect the basal *Cyp7a1 * transcription [44, 45]. However, the repressive effect of ciprofibrate on CYP7A1 mRNA expression and enzyme activity was completely abolished in mice lacking *Pparα*, providing an in vivo evidence that fibrates inhibition of *Cyp7a1 * was PPAR*α*-dependent [44, 45]. As mentioned earlier, CYP27A1 is the rate-limiting enzyme in the alternative bile acid synthetic pathway, and is also responsible for the side chain oxidation in the classic bile acid synthetic pathway (Figure 1). The *Cyp27a1 * transcription was also repressed by fibrate treatment in mice, despite a much weaker reduction in the mRNA level and enzyme activity when compared to those of *CYP7A1 * [45]. The fibrate inhibition of *CYP27A1 * was also likely to be PPAR*α*-dependent, but the molecular mechanism of this regulation is still not clear. Simultaneous inhibition of both bile acid synthetic pathways may result in decreased hepatic cholesterol catabolism and overall bile acid production in the liver. Unlike CYP7A1, which is specifically expressed in the liver, CYP27A1 is also expressed in peripheral tissues such as macrophages and intestines and is thought to play a role in cellular cholesterol efflux by converting cholesterol into oxysterols [46, 47]. It was found that *CYP27A1 * was upregulated by PPAR*γ* activation in human macrophages [48, 49]. A PPRE was identified in the human *CYP27A1 * promoter that specifically bound PPAR*γ*/RXR heterodimer. Treatment of a PPAR*γ* agonist caused an increased cholesterol efflux from human macrophages (Figure 3). Although how activation of PPAR isoforms led to tissue specific regulation of *CYP27A1 * in the liver and macrophages is not clear, these findings are in general consistent with the roles of PPARs in inhibition of overall hepatic bile acid synthesis and stimulation of reverse cholesterol transport (see Section 4.1).

The ratio of CA to CDCA in the bile determines the hydrophobicity of the overall bile acid pool in humans, and may affect biliary cholesterol solubility in the bile. Hydrophilic bile acid ursodeoxycholic acid has been used clinically to dissolve cholesterol gallstones [50]. CYP8B1 regulates CA formation in the classic bile acid synthesis pathway and plays an important role in controlling the CA : CDCA ratio. Clofibrate treatment has been shown to increase CYP8B1 activity and mRNA level in rat liver microsomes [51]. Treating mice a PPAR*α* agonist Wy14643 resulted in an up regulation of CYP8B1 mRNA levels and increased CA to CDCA/*β*-muricholic acid ratio, and knockout of *Pparα* reversed that [52]. A functional PPRE was identified in both mouse and rat *CYP8B1 * promoter, suggesting a direct transcriptional activation of *CYP8B1 * by PPAR*α*. Bezafibrate treatment has been shown to increase the CA to CDCA ratio in human patients, which further suggests that PPAR*α* regulation of CYP8B1 may likely be conserved in humans [40]. The observation that reduction in bile acid output by gemfibrozil in human was mainly a result of decreased CDCA in the biliary bile acid pool, while CA level was not significantly changed further suggested that PPAR*α* activation of CYP8B1 could compensate the reduction in overall bile acid synthesis and maintain CA levels after fibrate treatment [38]. Since increased CA to CDCA ratio may favor cholesterol solubilization, the direct induction of CYP8B1 by PPAR*α* may not contribute to the increased lithogenic index of the bile by synthetic PPAR*α* agonists. 

### 3.2. PPAR Regulation of Bile Acid Transport

Limited studies have implicated that PPARs may play a role in regulation of bile acid conjugation and transport in the liver and intestine. An early study showed that ciprofibrate feeding for two weeks resulted in a significant decrease of hepatic NTCP, OATP1 and BSEP in mice, and these effects were largely abolished in *Pparα* null mice [53]. This was supported by another study showing that down regulation of OATP and NTCP by perfluorinated fatty acids were PPAR*α*-dependent [54]. Consistent with decreased expression of hepatic bile acid transporters, biliary bile acid concentration was also decreased by ciprofibrate [53]. Although this study did not evaluate the biliary cholesterol saturation, the reported increase in bile flow and decreased biliary cholesterol concentration following ciprofibrate treatment seemed to contradict previous observations in humans. Further studies are required to evaluate the role of PPAR*α* in regulation of the hepatic and biliary bile acid transport systems. In the intestine, ASBT was found to be upregulated upon PPAR*α* activation by ciprofibrate in Caco2 cells [55]. The intestinal bile acid binding protein (I-BABP) was also found to be induced upon PPAR activation in Caco2 cells [56]. Upregulation of ASBT and I-BABP presumably increases intestinal bile acid uptake and intracellular transport. However, it is unclear how PPAR*α* may regulate intestinal OST*α*/OST*β* and basolateral efflux of bile acids. Mice lacking functional OST*α*/OST*β* heterodimer due to *Ostα* knockout showed significantly decreased bile acid pool and decreased serum bile acid concentrations [35]. Changes in bile acid concentration in hepatocytes and enterocytes may affect the activity of nuclear receptor FXR. FXR deficiency in the liver has been implicated in the gallstone formation in mice due to imbalanced expressions of cholesterol, bile acid, and phospholipid transporters [57]. Decreased basolateral bile acid efflux in *Ostα* null mice was associated with significantly decreased hepatic *Cyp7a1 * expression, likely due to induction of intestinal fibroblast growth factor 15 (FGF15), which inhibits *Cyp7a1 * expression via bile acid activation of FXR [35].

## 4. PPAR Regulation of Cholesterol Metabolism

Cholesterol is not only an essential cell membrane component for maintaining normal cell functions but also the precursor to all steroid hormones, bile acids, and oxysterols, which are important regulators in diverse metabolic pathways. High intracellular cholesterol is toxic to the cells, and high serum cholesterol built up in the arterial walls will lead to the plaque formation, one of the initial steps in atherosclerosis development. Hypercholesterolemia is considered as one of the leading causes of many cardiovascular and heart diseases and has become a major health concern worldwide. Fibrates are used to treat dyslipidemia mainly for its ability to stimulate fatty acid oxidation, while TZDs are used to improve insulin sensitivity and glucose homeostasis. It is suggested that PPARs may play a role in the development of atherosclerosis by modulating cholesterol metabolism as well as alleviating inflammation in the liver and vasculature [58]. The PPAR regulation of the pathways related to whole body cholesterol homeostasis will be summarized below.

### 4.1. PPAR and Reverse Cholesterol Transport

Plasma lipoproteins are macromolecules that carry triglycerides, cholesterol, and other lipids for tissue distribution and metabolism. In the blood circulation, cholesterol is carried on LDL and HDL particles. Studies in the past decades have linked elevated plasma LDL-C to higher risks of cardiovascular incidence. Thus, developing therapeutic agents that efficiently decrease plasma LDL-C has been a major pharmacological effort for the prevention and treatment for coronary heart diseases. So far, the use of HMG-CoA reductase inhibitor statins has consistently shown adequate reduction of plasma LDL-C levels by inhibiting the de novo cholesterol synthesis in the liver and increasing LDL receptor-mediated clearance of serum cholesterol [59–62]. However, even with an adequate control of plasma LDL-C, only an approximate 20–35% reduction in major cardiovascular events was seen in a randomized clinical trial [63]. In fact, a significant percentage of patients had normal plasma LDL-C levels at the onset of major cardiovascular events [64]. Early clinical trials have found that the risk of cardiovascular disease shows an inverse correlation with plasma HDL-C levels, and low HDL-C has been considered as a risk factor for cardiovascular diseases [65, 66]. Compared to the efficacy of statin therapies in lowering plasma LDL-C, no therapies have been established so far to raise plasma HDL-C, and current studies are in search for therapeutic agents that raise plasma HDL-C levels as a means to achieve further risk reduction of cardiovascular events in human patients.

The ability of plasma HDL in reducing the risk of coronary heart disease resides in its physiological function to transport excess cholesterol from peripheral tissues to the liver for excretion or reutilization, a process that is referred to as reverse cholesterol transport (Figure 3). The role of PPAR*α* in regulating HDL metabolism and promoting reverse cholesterol transport is supported by the clinical studies showing that fibrate treatments not only led to a marked reduction in plasma triglycerides but also caused about 5–15% increase in plasma HDL-C levels, with a modest reduction in LDL-C [67]. Accordingly, such induction of HDL-C can be translated into an approximately 25% reduction of the risk of coronary heart disease [68]. 

At the molecular level, fibrate effects on plasma HDL-C level are thought to be at least in part mediated by PPAR*α* induction of the Apolipoprotein AI (ApoA-I). ApoA-I and ApoA-II consist of the major protein moiety on HDL particles and serve as the receptor ligands for hepatic HDL uptake and metabolism [69]. HDL is initially synthesized by the liver and intestine. ApoA-rich and lipid-poor pre-*β*-HDL particles acquire cholesterol and phospholipids from peripheral tissues and circulating VLDL and chylomicrons, and then mature into HDL particles. Increased plasma ApoA-I by ApoA-I infusion or transgenic expression of *ApoA-I * were associated with increased plasma HDL-C and decreased atherosclerosis in experimental animal models [70–72]. PPAR*α* activation by fibrates induced ApoA-I mRNA expression in human hepatocytes [73]. A PPRE has been identified in the human *ApoA-I * promoter [74]. Interestingly, PPAR*α* effect on *ApoA-I * seems to be species-specific as PPRE is not conserved in rodent *ApoA1 * genes [74]. PPAR*α* activation in rodents actually decreased plasma HDL-C [73, 75], whereas genetic knockout of *Pparα* in mice showed increased ApoA-I mRNA expression and plasma ApoA-I and HDL levels [76]. Using human *ApoA-I * transgenic mice, Bertou et al. demonstrated that gemfibrozil increased hepatic human ApoA-I mRNA expression and plasma human ApoA-I and HDL levels [73], which provided an in vivo evidence that PPAR*α* activation positively regulates plasma HDL and reverse cholesterol transport in humans.

The ATP-binding cassette transporter A1 (ABCA1) is expressed in liver, intestine, and macrophages. ABCA1 plays a central role in HDL formation by transporting intracellular cholesterol to pre-*β* HDL particles (Figure 3). Both human patients with nonfunctional ABCA1 due to autosomal recessive disorder (Tangier disease) and ABCA1 knockout mice showed extremely low plasma HDL levels, underscoring the importance of ABCA1 in HDL metabolism [77, 78]. Several independent studies evaluating the PPAR effect on macrophage cholesterol efflux have found that both human and mouse ABCA1 are induced upon PPAR*α* and PPAR*γ* activation, suggesting PPAR may have an anti-atherogenic function by regulating cholesterol efflux from macrophages and thus reducing foam cell formation [79, 80]. However, no PPRE has been identified in *ABCA1*, and PPAR induction of *ABCA1 * expression may be an indirect effect. *ABCA1 * is a direct target of the oxysterol receptor, liver orphan receptor *α* (LXR*α*), which induces ABCA1 in response to high cellular cholesterol activation [81]. *LXRα* expression is induced by both PPAR*α* and PPAR*γ* agonists in human and murine macrophages [82]. In *Pparγ* knockout mice, both ABCA1 expression and cholesterol efflux were reduced in macrophages [80]. PPRE has been identified in both human and mouse *LXRα* promoter [82, 83]. Results from these studies supported a PPAR*γ*-LXR*α*-ABCA1 signaling cascade that mediates cholesterol efflux in macrophages. However, despite the critical role of cholesterol-laden macrophage in foam cell formation and development of atherosclerosis, it is believed that cholesterol efflux from macrophages may not contribute significantly to the total plasma HDL-C levels. Instead, liver and intestine represent the major sources of plasma HDL-C [84, 85]. PPAR*α* activation by Wy14643 has been shown to induce *Abca1 * expression in the mouse intestine [86]. However, as discussed in the next section, it seems that PPARs exert a negative effect on LXR*α*-dependent gene transcription in the hepatocytes via physical interaction with LXR*α* (next section). Thus, the relative contribution of this PPAR cascade in overall plasma HDL metabolism need to be further defined.

PPARs may also regulate several genes that are involved in HDL modification and metabolism. An important step in HDL metabolism is the cholesteryl ester transfer protein- (CETP-)mediated transport of triglycerides from VLDL and LDL to HDL in exchange for cholesterol esters. Mutations in *CETP * has been shown to increase plasma HDL levels with a modest reduction in LDL [87]. CETP is expressed in human but not mice. A recent study of human *CETP * transgenic mouse model showed that fenofibrate significantly reduced plasma CETP activity, which was correlated with elevated plasma HDL-C levels [88]. This study suggests that PPAR*α* activation may inhibit plasma CETP activity in humans and may contribute to elevated HDL-C by fibrate treatment. However, the association between CETP inhibition and cardiovascular risk reduction remains controversial, as clinical trials showed that although inhibition of CETP significantly increased plasma HDL levels, further reduction of atherosclerotic progression was not seen in patients receiving torcetrapib/atrovastatin combined therapy compared to patients receiving atorvastatin alone [89, 90].

Given the potential role of fibrates in raising plasma HDL-C, the statin and fibrate combined therapy has been tested in several clinical trials. In these studies, addition of fibrate significantly increased plasma HDL when compared to statin alone [91–93]. Certain fibrate/statin combination therapies were well tolerated by the patients, while others showed side effects. Larger trials are needed to further evaluate the benefit and safety for using fibrate and statin combined therapies in the treatment of hyperlipidemia.

### 4.2. PPAR and Cholesterol Synthesis

To elucidate the mechanisms of cholesterol lowering effect by fibrates, a limited number of studies have been carried out to investigate the effect of PPARs on hepatic de novo cholesterol synthesis. It was shown that feeding wild-type mice a diet containing the Wy14643 significantly decreased hepatic cholesterol synthesis rate, as measured by in vivo ^3^H_2_O incorporation. Such reduction in cholesterol synthesis was not seen in *Pparα* knockout mice [94]. Similar reduction of HMG-CoA reductase activity and hepatic cholesterol synthesis was also seen in rats receiving clofibrate treatment [95]. Consistent to these studies, PPAR*γ* agonist troglitazone has been shown to reduce cholesterol synthesis in hepatoma HepG2 cells and intestine Caco2 cells [96]. 

Recently, a few studies have indicated that the PPAR effect on de novo cholesterol synthesis may be mediated by PPAR-dependent inhibition of sterol response element binding protein-2 (SREBP-2) protein cleavage and maturation. SREBPs are transcriptional factors that regulate the expression of genes in cholesterol, fatty acid and triglyceride synthesis [97]. Three isoforms have been identified in mammals, SREBP-1a, SREBP-1c and SREBP-2. While SREBP-1 is believed to be mainly responsible for activation of genes in fatty acid and triglyceride synthesis, SREBP-2 preferentially stimulates genes in cholesterol synthesis and uptake, including HMG-CoA reductase and LDL receptor (LDLR). SREBPs are synthesized as a 120 KDa precursor protein that forms a complex with SREBP cleavage activating protein (SCAP) and is localized in the ER membrane. Upon sterol depletion, SREBP is translocated to the Glogi apparatus where a two-step proteolytic cleavage process releases a mature form of SREBP that enters the nucleus and activates gene expression by binding to a consensus SRE sequence in the gene promoters. The retention of SREBP/SCAP complex in the ER depends on its binding to the endoplasmic reticulum resident proteins, insulin-induced gene-1 (Insig-1), and Insig-2. Insig-1 and insig-2 are highly expressed in the liver [98, 99]. Increased Insig-1, but not Insig-2, was associated with increased endoplasmic reticulum retention of SREBPs under high sterol conditions [99]. Kast-Woelbern et al. first reported that a PPAR*γ* agonist rosiglitazone induced Insig-1 expression in the adipose tissue of diabetic db/db mice [100]. A functional PPRE was identified in *Insig-1 * promoter and binds PPAR*γ*. A similar induction in Insig-1, but not Insig-2, mRNA expression and a reduction of nuclear SREBP-2 by clofibrate was also reported in rats [101]. In a more recent study, Qin et al. showed that PPAR*δ* activation also induced Insig-1 in HepG2 cells [102], which correlated with a reduced amount of SREBP-1 mature form. The study by Qin et al. also showed that expression of PPAR*δ* in *db/db * mice by adenovirus-mediated gene transfer induced Insig-1 expression, inhibited SREBP-1c maturation, and alleviated hepatic lipogenesis. Although increased Insig-1 expression represses the cleavage of both SREBP-1 and SREBP-2, these authors did not observe reduced expression of SREBP-2 target genes including LDLR and HMG-CoA reductase, indicating PPAR*δ* may preferentially regulate SREBP-1c and hepatic fatty acid metabolism, but not cholesterol metabolism. It seems that three PPAR isoforms may regulate *insig-1* expression. However, since three PPAR isoforms exhibit different tissue expression profiles, activation of different PPAR isoforms by pharmacological agents may lead to somewhat distinct and tissue-specific effect on the activity of SREBPs, and thus fatty acid and cholesterol metabolism.

### 4.3. PPAR and Intestinal Cholesterol Absorption

Intestinal cholesterol absorption is thought to be coordinately regulated by Niemann Pick C1-Like1 protein (NPC1L1) and the ATP binding cassette half transporters ABCG5/G8 (Figure 3). NPC1L1 was first identified as a candidate gene for cholesterol transport based on its sequence homology to NPC1 [103]. NPC1L1 is highly expressed in the mouse small intestine. Genetic knockout of NPC1L1 in mice resulted in markedly decreased fractional cholesterol absorption [104]. In addition, fractional cholesterol absorption in *Npc1l1 * knockout mice was insensitive to the inhibition by ezetimibe, a potent cholesterol absorption inhibitor, suggesting that NPC1L1 plays a central role in intestinal cholesterol absorption. ABCG5 and ABCG8 are expressed on the canalicular membrane of hepatocytes and the apical membrane of the proximal small intestine. They form functional heterodimers and transport dietary plant sterols and cholesterol into the bile or intestine lumen. ABCG5/G8 were identified as the defective genes in a rare genetic disorder called sitosterolemia, where patients showed markedly increased plasma and organ plant sterol levels due to increased intestinal absorption and decreased biliary secretion [105]. Consistent with the proposed function of ABCG5/G8 in cholesterol transport, transgenic overexpression of ABCG5/G8 in mice caused a significant increase in biliary cholesterol secretion and decreased intestinal fractional cholesterol absorption [106].

Gemfibrozil or Wy14643 have been shown to inhibit intestinal cholesterol absorption in rats and mice [86, 107]. Similarly, a potent PPAR*δ* agonist GW610742 also reduces intestinal cholesterol absorption, which is correlated with decreased mRNA expression of NPC1L1 in the mouse intestine [108]. Recently, Valasek et al. showed that long-term fenofibrate administration inhibits NPC1L1 mRNA expression and fractional cholesterol absorption. These effects were abolished in *Pparα* knockout mice and further confirmed the role of PPAR in intestinal cholesterol absorption [109]. The molecular mechanism of PPAR inhibition of NPC1L1 is not clear, and is likely to be an indirect effect, secondary to changes caused by PPAR activation [109]. 

The mechanism of PPARs regulation of ABCG5/G8 is not known. Valasek reported that intestinal ABCG5 and ABCG8 are not involved in reduced cholesterol absorption in fenofibrate-fed mice [109]. In contrast, PPAR*α* was implicated in the fasting-induced hepatic ABCG5/G8 expression in mice [110].

## 5. Crosstalk of PPAR with Other Nuclear Receptors in Cholesterol and Bile Acid Metabolism

### 5.1. PPAR Crosstalk with LXR

The LXR subfamily of nuclear receptor consists of two isoforms: LXR*α* and LXR*β*. LXR*α* is expressed at high levels in liver, intestine and macrophages, while LXR*β* is universally expressed in most tissues. LXR*α* is activated by oxysterols such as 22(S)-hydroxycholesterol, 24(S), 25-epoxycholesterol, and 27-hydroxycholesterol, whose levels are thought to be proportional to cellular cholesterol levels [111, 112]. Extensive studies in the past have established LXR*α* as a central regulator of tissue cholesterol homeostasis by regulating a network of genes in cholesterol metabolism and excretion. In rodent, but not human livers, LXR*α* stimulates conversion of excess cholesterol to bile acids by activation of hepatic *Cyp7a1 * expression [113]. LXR*α* also stimulates the cholesterol efflux transporters ABCG5/G8 for biliary free cholesterol secretion [114]. In the intestine and liver, LXR*α* induces ABCA1 and ABCG1, which transport cholesterol to ApoA-I and HDL and thus promote reverse cholesterol transport [81, 115]. In macrophages, LXR*α*-dependent activation of ABCA1 and ABCG1 prevents cholesterol accumulation and atherosclerosis progression. Mice lacking LXR*α* are susceptible to high cholesterol diet induced hypercholesterolemia, while activation of LXR*α* by synthetic agonists show protective effects in hypercholesterolemic mice, demonstrating the critical role of LXR*α* in maintaining whole body cholesterol homeostasis [116–118]. However, the development of potent LXR*α* agonist for treating hypercholesterolemia was hindered due to the lipogenic effect of LXR*α* activation [119, 120]. Mice receiving LXR*α* agonists showed significantly increased hepatic fatty acid synthesis and elevated plasma triglyceride levels. It is now clear that the lipogenic effect of LXR*α* is mainly due to its transcriptional activation of SREBP-1c [121, 122]. 

PPAR*α* was identified as an interacting partner of LXR*α* in a yeast-two hybrid assay [123]. It has been shown that LXR*α* interaction with PPAR*α* blocked PPAR*α*/RXR heterodimer binding to the PPRE and resulted in inhibiting PPAR*α* target genes. Another study showed that PPAR*α*/LXR*α* interaction was enhanced by addition of an LXR*α* agonist TO901317, and PPAR*α*/LXR*α* interaction reduced PPAR*α*/RXR*α* heterodimer formation [124]. In mice fed TO901317, PPAR*α*-regulated genes in hepatic fatty acid oxidation were repressed suggesting that activation of LXR*α* may repress hepatic fatty acid oxidation via inhibition of PPAR*α* transcriptional activity. In contrast, LXR*α* activation induces PPAR*α* in mouse intestine [125, 126]. Further more, activation of LXR*α* by a specific agonist induced not only LXR*α* target genes but also PPAR*α* target genes in mouse intestine. As discussed in the previous section, PPAR*α* is also implicated in the intestinal cholesterol absorption and transport process. Thus, the identification of intestine-specific LXR*α*-PPAR*α* signaling cascade may provide an additional pathway for LXR*α*/PPAR*α* in coordinated regulation of cholesterol metabolism in the intestine.

The effect of PPARs on LXR*α*-dependent transcriptional network has also been studied, and both positive and negative effects have been reported. Polyunsaturated fatty acids (PUFA) inhibit hepatic lipogenesis by decreasing SREBP-1c mRNA and protein in cultured hepatocytes and animal livers [127, 128]. A study by Yoshikawa et al. suggested that activation of PPAR*α* caused decreased LXR*α*/RXR binding to the SREBP-1c gene promoter and resulted in down regulation of SREBP-1c and lipogenic gene expression [121, 129]. The finding that PPAR*α* activation inhibits SREBP-1c is in agreement with the known function of PPAR*α* in stimulating hepatic fatty acid oxidation and its lipid lowering effect in humans. Consistent with this notion, Matsusue et al. reported that activation of PPAR*δ* downregulated angiopoietin-like protein 3 gene in lipid metabolism via an LXRE on the angiopoietin-like protein 3 gene promoter [130]. However, these findings seem to contradict the existing reports that activation of both PPAR*α* and PPAR*γ* in macrophages induces LXR*α* gene expression and LXR*α*-dependent cholesterol efflux [79, 80, 130]. Because functional PPRE has been identified in both human and mouse LXR*α* gene promoter, the lack of activation of LXR*α* by PPARs in the liver is still not fully understood [82, 83]. In general, fibrate therapy showed protective effect against atherosclerosis in men. However, studies with fibrate administration in mice yielded mixed results. In hyperlipidemic LDLR knockout mice, activation of PPAR*α* or PPAR*γ* have been shown to prevent atherosclerosis and foam cell formation, and such protective effect seems to involve ABC-dependent cholesterol efflux pathways [131]. Similar antiatherogenic effects of PPARs were also found by studies using ApoE knockout mice [132, 133]. In contrast, genetic deletion of PPAR*α* in ApoE knockout mice resulted in more severe atherosclerosis [134]. Another study reported that ciprofibrate treatment in ApoE knockout mice promoted the progression of atherosclerosis [135].

### 5.2. PPAR Crosstalk with FXR

It is reported that bile acids, acting through nuclear receptor FXR, induced human PPAR*α* gene in HepG2 cells [136]. It is known that activation of FXR by bile acids or a synthesis FXR agonist negatively regulates hepatic fatty acid synthesis and plasma triglyceride levels [137]. The lipid lowering effects of bile acids are thought to be attributable to the inhibition of SREBP-1c activity in the liver. In concert, FXR induction of PPAR*α* may be an additional mechanism to antagonize hepatic SREBP-1c activity and promote hepatic fatty acid oxidation. However, the FXRE on the human *Pparα* is not conserved in murine *Pparα* gene promoter [136]. Consistent with this finding, mice fed a diet supplemented with bile acids antagonized PPAR*α* agonist effect [138]. It seemed that bile acid-activated FXR/SHP pathway was not involved in such regulation as bile acids still inhibited PPAR*α* activity in *Fxr * knockout mice [138]. Bile acid-activated cellular signaling pathways may be implicated in the negative regulation of PPAR*α* in mice. SHP is generally considered as a negative regulator by interacting with other nuclear receptors and transcriptional factors. Both PPAR*α* and PPAR*γ* physically interact with SHP [139, 140]. Surprisingly, both studies found that SHP was able to enhance PPAR*α*- and PPAR*γ*-mediated transcriptional activity. The study by Nishizawa et al. showed that SHP competed with corepressrors for the binding to PPAR*γ*, which provided a possible explanation of SHP effect on the transcriptional activity of PPARs [140]. As SHP does not possess intrinsic transcriptional activity, the positive effect of SHP on PPAR transcriptional activity was somehow unexpected, and the physiological relevance of the role of SHP in regulating PPAR signaling needs to be further defined in future studies.

## 6. Conclusion and Future Perspectives

In the past decades, the roles of PPARs have been extended from stimulating fatty acid oxidation and glucose metabolism to regulating cholesterol and lipoprotein metabolism, bile acid metabolism, energy homeostasis and inflammation, and so forth. Currently, most of the regulatory roles of PPARs turned out to be beneficial in improving dyslipidemia and glucose homeostasis and reducing the risks of major cardiovascular and heart events, while others may represent adverse effects associated with the use of certain PPAR agonists. With the ability of PPARs to crosstalk with other protein factors and cellular signaling pathways, it is not surprising that more regulatory roles of PPARs have been revealed. Long-term fibrate therapy represses hepatic bile acid synthesis and increases the incidence of cholesterol gallstone. However, the role of PPAR in bile acid transport are still not clear. The use of PPAR agonists for treating steatohepatitis has also been considered due to their known effects in fatty acid metabolism, inflammation, and hepatic fibrosis [141–145]. In addition to the repressive effect on hepatic bile acid synthesis, PPAR*α* have recently been implicated in the positive regulation of bile acid conjugation and toxicity [146–149]. Whether these regulatory roles of PPAR*α* represent any beneficial effects in cholestatic liver injury needs to be further explored. PPAR agonists have been proven to be a group of drugs with great therapeutic potentials in treating metabolic syndromes. Clinical trials are being conducted to evaluate the efficiency and safety of fibrate/statin combined therapy. Given the increasingly recognized epidemic of obesity, diabetes, and chronic liver diseases associated with metabolic disorders, more potent and selective PPAR agonists need to be developed to achieve desirable biological effects and to avoid adverse effects. The development of such agents will depend on a better understanding of the regulatory roles of PPARs in diverse biological processes beyond triglyceride and glucose metabolism.
